# Tick-Borne Surveillance Patterns in Perceived Non-Endemic Geographic Areas: Human Tick Encounters and Disease Outcomes

**DOI:** 10.3390/healthcare9060771

**Published:** 2021-06-21

**Authors:** Sarah P. Maxwell, Connie L. McNeely, Kevin Thomas, Chris Brooks

**Affiliations:** 1 Economic, Political and Policy Sciences, The University of Texas at Dallas, Richardson, TX 75080, USA; 2 School of Policy and Government, George Mason University, Fairfax, VA 22030, USA; cmcneely@gmu.edu; 3 Laboratory for Human Neurobiology, Boston University School of Medicine, Boston, MA 02118, USA; kipthoma@bu.edu (K.T.); crbrooks@bu.edu (C.B.)

**Keywords:** tick bite encounters, tick-borne disease, surveillance, patient reports, non-endemic

## Abstract

Recent scholarship supports the use of tick bite encounters as a proxy for human disease risk. Extending entomological monitoring, this study was designed to provide geographically salient information on self-reported tick bite encounters by survey respondents who concomitantly reported a Lyme disease (LD) diagnosis in a state perceived as non-endemic to tick-borne illness. Focusing on Texas, a mixed-methods approach was used to compare data on tick bite encounters from self-reported LD patients with county-level confirmed cases of LD from the U.S. Centers for Disease Control and Prevention (CDC), as well as serological canine reports. A greater proportion of respondents reported not recalling a tick bite in the study population, but a binomial test indicated that this difference was not statistically significant. A secondary analysis compared neighboring county-level data and ecological regions. Using multi-layer thematic mapping, our findings indicated that tick bite reports accurately overlapped with the geographic patterns of those patients previously known to be CDC-positive for serological LD and with canine-positive tests for *Borrelia burgdorferi*, anaplasmosis, and ehrlichiosis, as well as within neighboring counties and ecological regions. LD patient-reported tick bite encounters, corrected for population density, also accurately aligned with official CDC county hot-spots. Given the large number of counties in Texas, these findings are notable. Overall, the study demonstrates that direct, clinically diagnosed patient reports with county-level tick bite encounter data offer important public health surveillance measures, particularly as it pertains to difficult-to-diagnose diseases where testing protocols may not be well established. Further integration of geo-ecological and socio-demographic factors with existing national epidemiological data, as well as increasingly accessible self-report methods such as online surveys, will contribute to the contextual information needed to organize and implement a coordinated public health response to LD.

## 1. Introduction

Vector-borne diseases, caused by viruses, bacteria, and parasites transmitted to humans by intermediary organisms (vectors), such as ticks and mosquitoes, have become significantly more widespread in the United States (U.S.) over this past decade [1]. Lyme disease (LD) is one such affliction and is the fastest growing vector-borne disease in the country. The total number of new LD cases in the U.S. per year is reported to exceed 300,000 [2,3,4]. However, the number of LD patients with lingering symptoms was projected to be as high as 1,944,189 in 2020 [5]. LD is spread by bites from blacklegged ticks (*Ixodes scapularis*), also called deer ticks, and has been linked specifically to the pathogenic bacteria *Borrelia (Borreliella) burdorferi* [6].

LD, especially in later stages, is difficult to diagnose, and variations in symptoms that present across patients can present challenges for both doctors and patients—for example, some patients may present the classically identifying erythema migrans rash (“bullseye” rash) and others may not. Moreover, LD is a multi-system condition that can manifest quickly and, furthermore, may produce symptoms that are often mistaken for other diseases such as influenza or various auto-immune disorders [2,7]. Late-stage LD is known to cause a host of lingering and debilitating symptoms, such as cardiac, rheumatological, or neurological manifestations [8]. “To qualify for the diagnosis of LD, patients must have Lyme-compatible symptoms and signs that are either consistently or variably present for six or more months” [9]. However, the multitude of symptoms, including psychological components, may interfere with diagnosis. In fact, LD is often used as a catch-all term for a set of complex concurrent infections.

Current human LD surveillance involves cases that meet CDC serological criteria and are reported to public health agencies. Importantly, no agency or organization tracks cases of Lyme disease patients who do not meet CDC criteria. The CDC publishes LD cases by county, some of which may have been acquired during travel and not locally acquired in the county of diagnosis. If clinically diagnosed patients receive tick bites in the same counties as those who meet official public health records, defined as locally acquired cases, further investigation is indicated to determine geographic overlap in other states, which can be a critical diagnostic indicator. This study demonstrates and fills this gap in one region by analyzing local health data and excluding those cases that limit the usefulness of CDC official reports. It is possible that some patients diagnosed with LD also will have co-infections from other tick-borne diseases (TBDs). The study also employs a comparison to canine ehrlichiosis, LD, and anaplasmosis, as the CDC and many states do not provide human TBD data at the county level for use in research or analysis.

Given the problematic diagnostic indicators and lack of county-level tick-borne disease data available to researchers and the public health community, the following research questions are proposed. (1) Can self-reported patient experiences inform public health policy and offer insights into illnesses with limited diagnostic support? (2) Can mapping patient self-reports of tick bites who also report clinical or serological diagnosis serve as a promising methodological approach to disease surveillance? Moreover, these questions are encompassed in a more overarching and fundamental research consideration: (3) Can thematic multi-layer mapping of LD patients with tick bite location recall serve as a proxy for human disease risk in areas often perceived to be non-endemic?

The overall purpose of this study is to determine if clinically diagnosed and CDC-positive LD cases are geographically similar in disaggregated form via county, bordering county, and ecosystem. Comparisons between patient self-reported disease and official counts of disease are not widely used methods of surveillance, but are important epidemiological tools when disease can be linked to an event, such as a tick bite. Recent research on endemic states indicates that “self-reported human tick encounters are a robust surrogate for human tick-borne disease associated with *Ixodes scapularis* at the household and individual levels” [10].

As the regional focus for the study, Texas was selected given its large size and diverse climate and ecological conditions, reflecting many of the environments supportive of tick presence and breeding. The CDC confirms that LD cases are known in Texas, and there are reported and established *I. scapularis* ticks. The size and vast number of counties in the state provide a geographic picture in which overlap of tick bite encounters and reported LD diagnosis with official reports are unlikely to be random coincidence. Texas, along with some other states, has been referred to as “non-endemic” [11,12], indicating the perception that it is a low-risk area for human tick-borne disease. However, confirmed cases with the Texas Department of State Health revealed specific clusters of LD in Texas [11]. An earlier study “found large numbers of chronically ill *Borrelia burgdorferi* PCR and sero-positive patients in Houston, Texas, a zoonotically ‘non-endemic’ area” [13]. Another study notes the presence and spread of the tick vector *Ixodes scapularis*, with a model that “indicates a wide distribution for *I. scapularis*, with higher probability of occurrence along the Gulf of Mexico coast. Results of the modeling approach predict that habitats suitable for the distribution of *I. scapularis* in the Texas-Mexico transboundary region will remain relatively stable until 2050” [14]. Another recent study found that patients with LD in Texas had similar and equally severe LD symptoms, whether diagnosed clinically or serologically [15]. Headaches, extreme fatigue, gastrointestinal manifestations, brain fog, neck and back pain, and influenza-like symptom effects on quality of life were found to be “powerfully negative” for both diagnostic groups [15]. Similar reports can be found among patients with lingering symptoms who were not serologically positive, but had symptoms significantly more severe than the control group [16].

This study aims to highlight the importance of spatial analyses among multiple indicators to improve disease surveillance and knowledge of human risk. Suggestive findings of LD and/or another TBD with known tick bites in non-endemic areas also highlight the need to pursue further surveillance and possible follow-up testing of patients. Extending this notion is surveillance by ecosystem, as ecosystems are not subject to smaller boundaries, such as county lines. A finding of self-reported LD cases (via clinical or serological diagnosis), supported by known tick bites in CDC-positive ecological regions (eco-regions), provides an indicator that patient-reported LD and CDC-confirmed cases are strongly tied. This study uses county level, human, canine, and ecological data, providing an investigative snapshot of geographic overlap in Texas.

## 2. Materials and Methods

This study explores how patients are diagnosed with LD, specifically via their tick bite encounters while experiencing a self-reported diagnosis of a tick-borne disease. Analytical focus is on cases in Texas, as research on states with lower levels of LD prevalence are limited.

### 2.1. Texans and Ticks (TTS) Survey

Data for this study were drawn from a specialized survey—the Texans and Ticks Survey (TTS)—developed to collect state-, county-, and zip-code-level LD self-reported patient information. TTS included the geographic location of tick bite incidents (if any). Survey participants were visitors to a social media site entitled “Texans and Ticks”, providing educational and prevention information. Open for a year, the site garnered more than 50,000 unique visitors. Designed to attract Texan residents with LD experience, it provided a convenience sample for survey administration. Visitors to the site were offered an anonymous link to participate in the survey, which was active for six months in the last half of 2018. Survey data were collected from the site using Qualtrics, an online survey platform. TTS participation was voluntary and respondents were required to be 18 years or older in age, although it was possible for an adult family member to complete the survey on behalf of a child. All responses were anonymous, and data were available only via a password-protected secured site.

All survey respondents reported an LD diagnosis by a medical professional. Survey respondents were asked if they had received an LD diagnosis, and if so, how they had been diagnosed. They could select: (1) Clinically (the doctor thinks you have Lyme Disease based on your symptoms); (2) Western Blot, where some bands were positive; (3) Western Blot, where five or more bands were positive (“CDC-positive”); (4) IGeneX or other specialty lab; or (5) I do not know/Not sure. Response choices were based on the CDC-recommended two-test serologic approach involving an enzyme-linked immunosorbent assay, followed by a Western blot test for specimens yielding positive or equivocal results [17]. The Western blot test identifies individual antigen patterns or “bands” typically seen in *Borrelia burgdorferi*, and the CDC requires identification of 5 or more bands out of 10 to be positive for LD diagnosis. However, reliance on this approach can be seen as problematic due to variations in patient sensitivity at different stages, and clinical diagnoses may involve broader perspectives and more comprehensive testing, as has been offered by various specialty laboratories, such as IGeneX, well known for its work with TBDs. Respondents who selected “I don’t know/Not sure” were excluded from the analysis. Survey respondents were also asked if they recalled a tick bite encounter, and, if so, to provide the county or zip code where the bite occurred. Only the respondents who reported an LD diagnosis and provided tick bite location were included in the analysis.

#### TTS Respondent Tick Bite Encounters

Respondent tick bite encounters of those who self-reported an LD diagnosis were mapped at the county level. Since counties with higher populations would naturally experience more tick bites (all else held constant), by-county raw case frequencies were corrected with respect to the county’s population density and standardized as the number of cases per 100,000 individuals. One study aim was to assess matching criteria among respondents to CDC official LD data, and geographic location of known tick bites was particularly important in light of responses indicating clinical diagnostic criteria.

### 2.2. Multi-Layer Thematic Mapping and Statistical Analysis

TTS information allowed for comparative analysis of locations to CDC-confirmed LD cases in Texas, canine cases of other tick-borne co-infections, and studies confirming cases or tick testing in various Texas ecoregions. TTS data were mapped by county, presented comparatively by county maps with CDC and canine reports to demonstrate matching and overlaps using the Excel mapping function. The CDC data include LD human case reports from 2000 to 2018. Case report definitions changed in 2008 to include “confirmed” and “probable” cases [17]. Canine data were developed from an online, public mapping system of veterinary lab reports [18]. The raw data are not available to researchers, but the online mapping reports are pulled from IDEXX Laboratories and Antech Diagnostics, which are the two largest veterinary labs in the United States. A Chi-Square Goodness-of-Fit test was also conducted on the reported age categories to determine if their frequency was unequally distributed. Additionally, a binomial test was conducted to determine if a greater proportion of respondents reported recalling a tick bite.

#### 2.2.1. Comparison Mapping of TTS Respondents to Human Lyme Disease Cases

Recognizing that some patients never develop the classic erythema migrans rash, reliable identification of LD symptoms is a key issue [19]. Concern about ticks leads to improved protective behaviors [20], indicating the need for further understanding and prevention. In non-endemic states, residents may be less likely to worry, and hence less likely to look for ticks or a rash after outdoor activity. “In these cases [sans a rash], the primary manifestations of acute illness include flu-like or viral-like systemic patient-reported symptoms such as fever, chills, malaise, fatigue, generalized achiness, and head and neck pain” [21]. Symptoms such as chills, fever, and neck pain are often not recognized as LD [22]. LD and other tick-borne pathogens are reported by patients and are also known to cause gastrointestinal manifestations, such as anorexia, abdominal pain, nausea, and vomiting [15,23]. Importantly, different types of ticks carry different pathogens, making geography and related conditions central to chronic illness knowledge, as symptoms may present differently from one part of the country to another depending on possible co-infection (e.g., Ehrliciosis, Babeosis, or Anaplasmosis) [24]. Few studies offer information regarding direct patient-provided data, which may serve as a proxy regarding tick populations and disease. For example, reporting state-level data, one study sampled ticks in Texas from 2008 to 2014, finding thirteen different tick species and noting that 23% of ticks collected carried disease pathogens [25].

#### 2.2.2. Comparison Mapping of TTS Respondents to Canine Tick-Borne Diseases

Canine reports were also analyzed in comparison to survey respondent tick bite encounters. In states such as Texas, where the CDC indicates the presence of LD, ehrlichiosis, and numerous other TBDs, but the actual number of cases may be unknown, it is possible to refer to veterinary studies that capture tick-borne illness as a basis for comparison [16]. Evidence of antibodies for *Borrelia burgdorferi*, genus *Ehrlica*, and *Anaplasma phagocytophilum,* among other tick-borne co-infections, has been found in canine samples across the entire southeastern United States and extends westward through Texas [26,27]. For canines, *Borrelia burgdorferi* were not considered endemic to Texas, yet were present in clear focal areas at the county level [26]. Veterinary studies continue to suggest that monitoring evidence of canine exposure to *Lyme borreliosis* and other tick-borne diseases could “allow early recognition of geographic expansion of endemic areas as well as identify hyper-endemic areas where both humans and dogs are at increased risk of infection” [28]. This study mapped canine TBDs for comparison.

Table 1 from the CDC presents an overview of *Ixodes scapularis* ticks, pathogens, and associated disease. This information was used to inform the disease mapping overlays in this study.

#### 2.2.3. Comparison Mapping of TTS Respondents by Neighboring County and Eco-Regions

County data were compared by Lyme or tick-borne disease reports, by neighboring county evidence, and by overlapping evidence within Texas eco-regions, as designated by the Texas Parks and Wildlife Department [29]. Neighboring counties were selected in relation to possibilities of LD or tick-borne illness in the immediate geographic area.

Looking to affected individuals for relevant detailed information on their experiences can contribute to improved diagnostic accuracy. This study takes an important methodological step in analyzing evidence derived from patient surveys, along with other data, for LD surveillance and tracking.

## 3. Results

The survey produced 111 full responses, with 95 respondents reporting having LD (Table 2). Forty-five percent of respondents with a self-reported LD diagnosis recalled a tick bite, resulting in 22 Texas counties.

Data from 16 respondents who reported not having LD were excluded from the analyses. All TTS respondents in this analysis reported a diagnosis of LD. TTS respondents were primarily under the age of 60 (91%); were working indoors (33%); were students (21%); or were not working (34%).

### 3.1. TTS Respondent Demographics, Symptoms, and Diagnostic Information

Data were analyzed for common patterns and total responses. As an initial step and extending the sample profile, baseline information was reviewed on how TTS respondents were diagnosed as LD positive, especially in the face of diagnostic difficulties and controversies. Respondents could select “all that apply” from a list of non-CDC-positive options, or select CDC serologically positive. Non-CDC-positive choices included clinically diagnosed, some Western Blot bands positive, and specialty laboratories.

Patients who did not meet CDC guidelines but experienced tick bites and LD symptoms will not appear in official healthcare or surveillance data, hindering geographic comparison. Almost half of TTS respondents reported an LD clinical diagnosis by a medical professional. Respondents were able to select more than one diagnostic criteria, suggesting that specialty laboratories or “non-CDC-positive” Western blots may have been considered in the clinical diagnosis, or played a confirmatory role following a clinical diagnosis. Twenty-three percent met the five-band criteria as CDC-positive cases. Forty-one percent of respondents with self-reported LD had confirmed LD through IGeneX or other specialty laboratories.

In this study, TTS respondents overwhelmingly reported symptoms such as extreme fatigue, neck and back pain, headaches, night sweats, depression, anxiety, brain fog, and persistent influenza-like symptoms. Indeed, almost 90% of respondents were plagued by extreme fatigue and almost 85% had ongoing influenza-like symptoms, consistent with patients who are CDC-positive for LD.

### 3.2. Multi-Layer Thematic Mapping of TTS Respondents to Human Lyme Disease Cases

**Figure 1:**
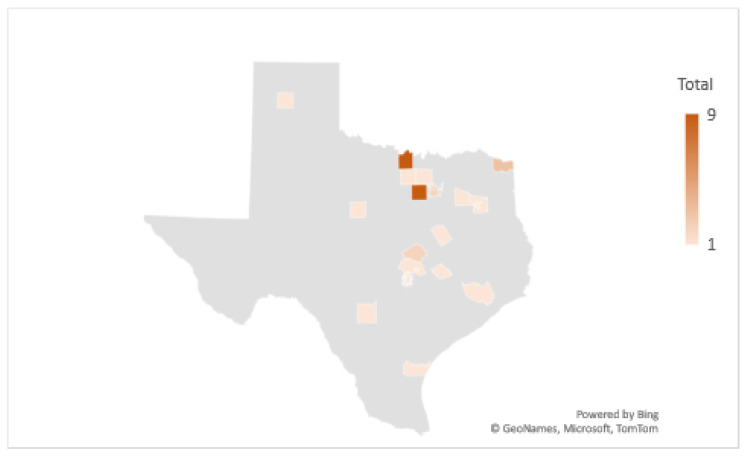
TTS respondent tick bite reports, total by county.

Using official data from the CDC, human LD patterns from 2010 to 2018 were included for visual comparison [17]. Figure 1 shows a total of 179 LD tick bite encounters in 22 counties out of a possible 254 in Texas. Counties with TTS self-reported tick bites were grouped by the number of reported tick bite encounters and a map report was created to provide information regarding self-reported LD cases and tick bite encounters in Texas (Figure 1). CDC-positive respondents who reported a tick bite in a Texas county were included in the analysis, as the CDC cases overlapped with all other reported counties by non-CDC-positive diagnostic type. Importantly, the comparison map and the county list were consistent in indicating counties in which LD respondents actually received tick bites. For example, one case of CDC-positive LD is reported in Limestone County, but the patient reports receiving a tick bite outside of the county of residence (i.e., Limestone). All counties were reviewed for locally acquired reports. Counties in which a patient was not infected, but resided, were considered non-LD official counties for comparison. Figure 1 shows the total number of tick bite encounters for each reported county.

**Figure 2:**
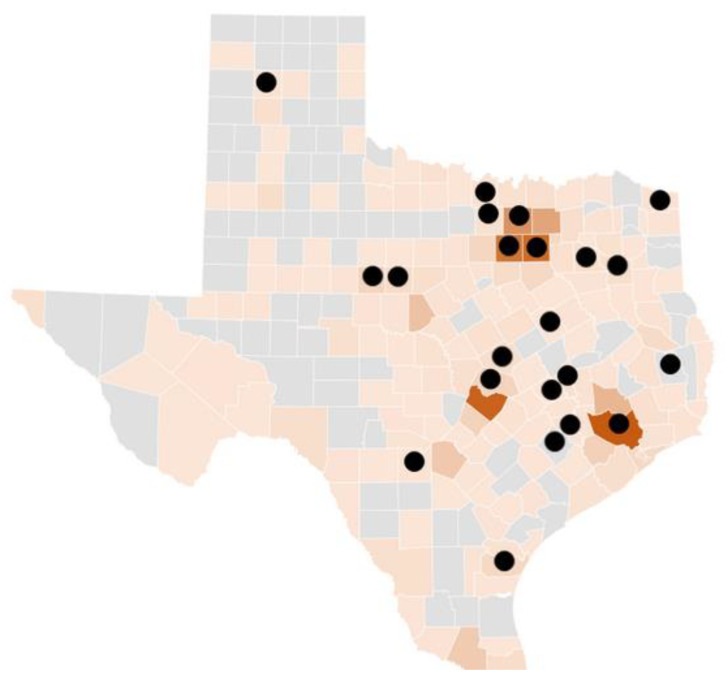
CDC human case reports from 2000 to 2018 with overlapping TTS respondent tick bite recall by counties in Texas.

Figure 2 combines CDC official human LD cases with the TSS tick bite encounters to provide a multi-layer thematic map. Figure 2 overlays TSS respondents onto the CDC-positive county map, which includes 173 counties. Each TTS dot represents one county. Multiple tick bites may have been reported in one county, and, therefore, one dot may represent multiple TSS tick bite encounters that overlay on a shaded CDC county.

In the baseline analysis comparing TTS self-reported cases to CDC-positive maps, of the 22 counties in which TSS respondents reported tick bites, 19 mapped exactly to CDC-positive cases. Overlap in 19 of the counties is noteworthy given the lower incidence rates of LD and other TBDs in Texas as compared to more endemic states.

TTS tick bite reports by county were also compared to bordering counties, as LD and other tick-borne illnesses do not necessarily adhere to county lines. All TTS-respondent counties had neighboring counties with known CDC-positive cases (Figure 2). To the best of our knowledge, this is the first account of self-reported tick bite encounter comparisons to CDC-positive cases, as well as LD county-level hot-spot clusters. Additionally of note, TTS respondents did not report tick bites in county clusters where the CDC reports zero human cases, i.e., portions of West Texas and the Panhandle.

A Chi-Square Goodness-of-Fit test also indicated that there was a significantly unequal distribution of respondents across age categories (Chi2 Statistic = 28.21; *p* < 0.001; df = 4). Although a greater proportion of respondents reported not recalling a tick bite, a binomial test indicated that this difference was not statistically significant (*p* = 0.4119).

**Figure 3:**
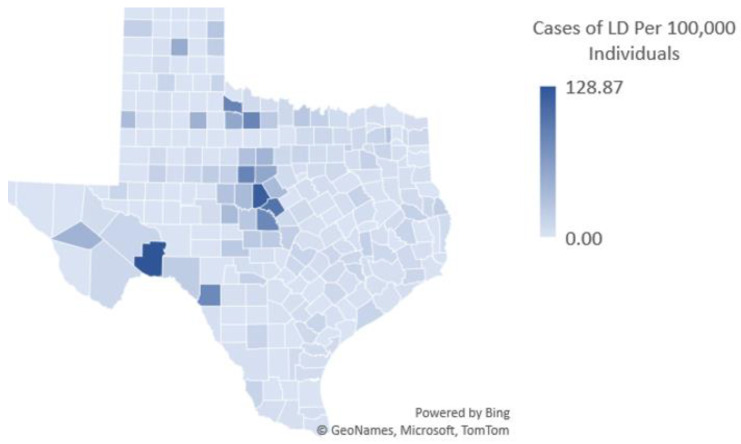
CDC Lyme disease case reports from 2000 to 2018 by population density.

Overall, the counties with the most locally acquired CDC cases are: Harris (179); Travis (169); Dallas (156): Tarrant (145); Denton (107); Collin (83); Montgomery (61); Brown (46); and Bexar (38). The latter three counties are also endemic, but are not highly populated, indicating that LD and tick bite recall is not simply a factor of population size. To demonstrate, a population density map of CDC human Lyme disease cases is presented in Figure 3. Figure 3 shows that cases are not simply a result of more populated areas.

### 3.3. Multi-Layer Thematic Mapping of TTS Respondents to Canine Tick-Borne Disease Cases

**Figure 4:**
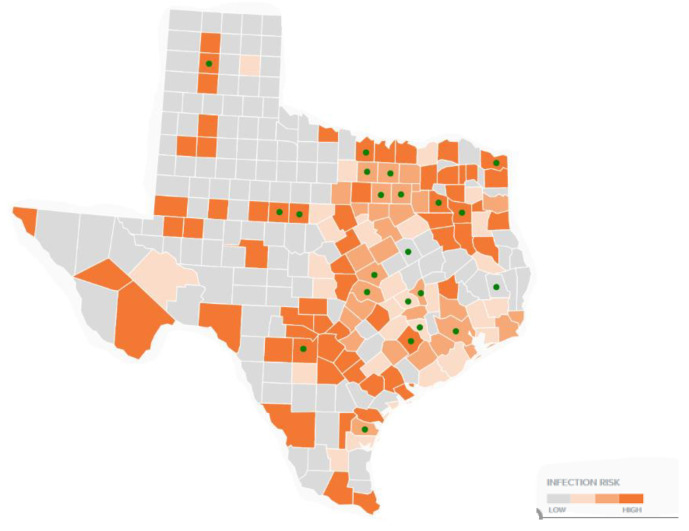
2018 canine ehrlichiosis and TTS respondent tick bite encounters. (Data Source: Companion Animal Parasite Council, Ehrlichiosis Canine Report, 2018; Raw data source, IDEXX Laboratories and Antech Diagnostics.)

**Figure 5:**
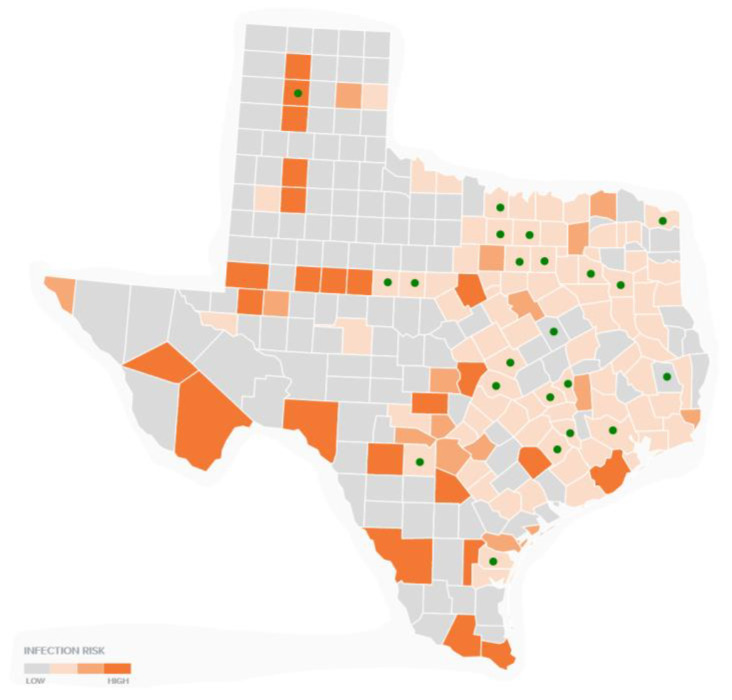
2018 canine anaplasmosis and TTS respondent tick bite encounters. Data Source: Companion Animal Parasite Council, Ehrlichiosis Canine Report, 2018; Raw data source, IDEXX Laboratories and Antech Diagnostics.

**Figure 6:**
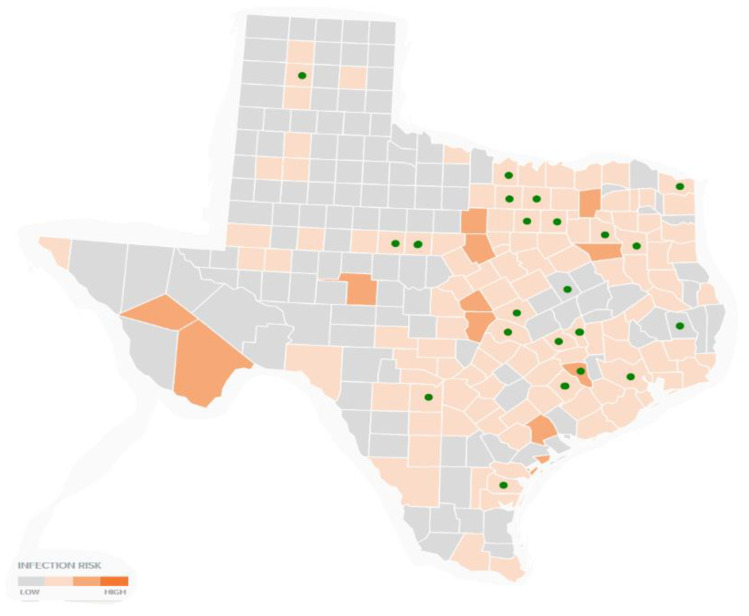
2018 canine Lyme disease and TTS tick bite encounters. Data Source: Companion Animal Parasite Council, Ehrlichiosis Canine Report, 2018; Raw data source, IDEXX Laboratories and Antech Diagnostics.

In addition to information from human CDC reports, canine studies on LD, i.e., Borrelia burgdorferi and co-infections, anaplasmosis, and ehrlichiosis, were included for comparison mapping (Figure 4, Figure 5 and Figure 6). TSS survey respondents who self-reported an LD diagnosis and also recalled a tick bite were mapped over canine cases, which were confirmed through serological testing. Figure 4, Figure 5 and Figure 6 provide multi-layer thematic maps demonstrating overlapping evidence of self-reported LD and tick bites to canine disease, since LD is often used as a catch-all term for numerous TBDs that cause similar symptoms.

Figure 4 provides an overlay of TTS respondents and canine ehrlichiosis. Twenty of the 22 counties overlap. No TTS respondent with LD and a tick bite encounter reported tick bites in counties in cluster areas absent of canine ehrlichiosis.

Figure 5 offers TTS respondent county overlap with canine anaplasmosis confirmed cases. Twenty out of the twenty-two counties map exactly, and county clusters absent of anaplasmosis also received zero tick bite reports by TTS respondents.

Figure 6 provides a multi-layer thematic map of TTS respondents over counties with positive LD canine reports. As with other canine tick-borne disease reports, twenty out of the twenty-two counties map exactly, and county clusters absent of canine LD also received zero tick bite reports by TTS respondents. LD canine confirmed cases appear in counties that do not have CDC-confirmed human case reports, indicating that LD is possibly present in counties unknown to the CDC.

### 3.4. Multi-Layer Thematic Mapping of TTS Respondents to Eco-Regions and Precipitation Suitable for Tick Populations

**Figure 7:**
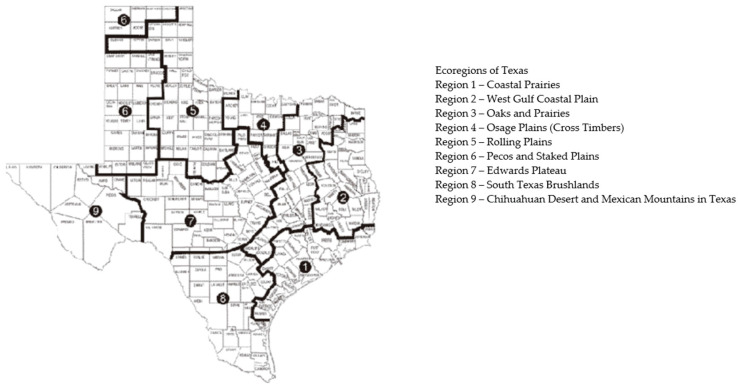
Eco-regions of Texas.

Extending the analysis to eco-regions, TTS tick bite reports align with serological canine statistics, and are *not* found in eco-regions six and nine among TTS respondents (Figure 7). Eco-regions six and nine in Texas also contain very few official canine cases, an important overall finding, as the Texas eco-regions produce varied patterns that point to unusual comparisons. For example, eco-region five, Rolling Plains, extends into the Texas Panhandle, encompassing a few counties that border eco-region six, an area with very limited case reports, human or canine. Potter County, as one outlier example, neighbors eco-region six, but contains geography and moisture levels distinctive to eco-region five and, therefore, appears in canine and human analysis as ripe for ticks and LD. Recent scholarship suggests that the regions where LD respondents report ticks are suitable for *I. scapularis* tick populations, which occur primarily in Eastern and Central Texas [29].

All TTS cases overlapped in seven out of the nine Texas eco-regions, including Potter County in eco-region five. Given the near zero CDC cases found in the western eco-regions six and nine, Pecos and Staked Plains and Chihuahuan Desert and Mexican Mountains, the lack of clinical and self-reported LD cases in those eco-regions is important. The majority of *I. scapularis* are found in the overlapping eco-regions, including the Coastal Prairies [30]. TTS respondents follow the same patterns.

*I. scapularis* is found in shrubs, a variety of forests, and grasslands throughout the U.S., including Texas. The southern states provide additional hosts on which deer ticks feed, such as lizards. Precipitation plays a role in tick habitats.

**Figure 8:**
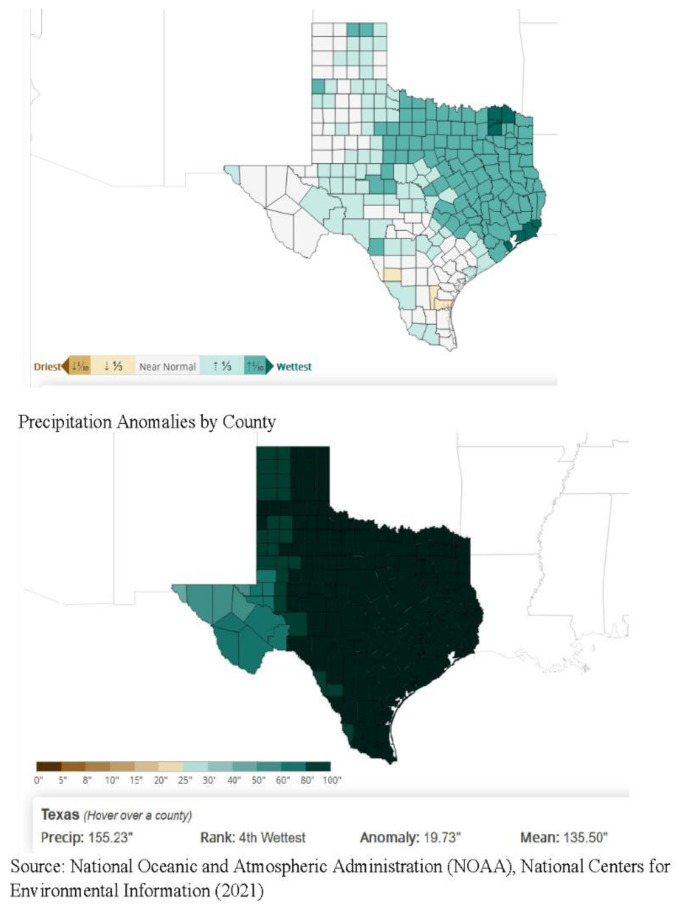
Annual precipitation and precipitation anomalies by Texas county, 2015–2020, month of July.

Figure 8 offers precipitation comparisons by county in Texas over the last five years for the month of July, a time when ticks are highly active. These counties also overlap with TSS, canine, and CDC cases.

TTS findings overlap with multiple counties in the Cross Timbers area, including Montague, Wise, Denton, Tarrant, and Callahan counties. Additionally, the tick encounter map distinctly overlaps in key county patterns relative to veterinary research showing *Borrelia burgdorferi*, *Ehrlichia canis*, and *Anaplasma phagocytophilumin* in Texas canines [26]. TTS showed that canine tick-borne diseases generally clustered in the same or neighboring counties, with important implications for understanding co-infection patterns. These results indicate that LD and co-infections may be present in distinct geographic patterns in Texas.

## 4. Discussion

### 4.1. Significance of Findings

Overall, the findings suggest distinctive patterns of overlap, indicating patients who are diagnosed clinically or via specialty laboratories, and indicating tick bite encounters in the same locations where human and canines report official serological cases. Clinically diagnosed patients are not counted by health departments, resulting in a lack of research on the geographic locations of LD patients who do not meet CDC diagnostic criteria. This study suggests that symptoms indicative of a tick-borne illness, with a tick bite recall, and a clinical or specialty lab diagnosis, may serve as a proxy for human disease risk, especially in areas perceived as non-endemic.

This study provides promising approaches to assessing tick-borne human disease risk in areas perceived to be non-endemic to ticks and disease. Mapping patient illness with concomitant tick bite recall in comparison to known indicators of tick-borne disease can serve as a proxy for establishing human risk. An important finding in this study indicates that counties, such as Potter in the Texas panhandle, where tick bites and disease are reported might have otherwise been considered inhabitable for ticks. Potter County happens to exist as a small extension of eco-region 5, where precipitation is heavier and habitats are more suitable for ticks.

A collective understanding in public health of LD and other tick-borne disease conditions and prevalence remains incomplete. Integrated approaches (e.g., the One Health Model) recognize that human, animal, and environmental relationships are integral to public health, particularly with respect to zoonotic diseases [31]. Spatial cluster analyses have shown promise in consolidating variable symptoms and incidence [32] and, as such, geographical considerations for determining tick presence and density are recognized as key factors for quantifying LD prevalence. Recent research supports the use of tick encounters as a proxy for LD and TBD risk [9].

This study takes an important step in analyzing evidence derived from patient reports for LD surveillance and tracking in a non-endemic area. The match between CDC-positive counties with the highest cases and those of TTS self-reported cases is remarkable. It is not simply the overlap of counties, but the severity of “hot-spot” LD county clusters; county clusters are the same for patient self-reported tick bites and CDC-positive reports, with cases that are not locally acquired removed from the CDC LD case map. These findings are especially notable given the limited number of CDC-confirmed cases in Texas.

Primary care physicians may under-diagnose LD in areas perceived as non-endemic [33]. Additionally, misdiagnosis was reported in seventy-two percent of respondents in a large survey [34], indicating the need for improved surveillance beyond entomology that links tick encounters with human disease risk, which can inform diagnostic approaches. Accordingly, the need for expanded and improved LD research and knowledge is highly apparent for the benefit of both patients and health practitioners.

Given that LD is often labeled a “contested illness,” TTS respondents who may be perceived as “faking it” could easily report any random county if their tick bites were indeed a false entry in the TTS survey. In other words, it would be highly unlikely that the totality of respondents’ tick bite reports would map directly to confirmed official CDC cases or canine serological findings through attempted deception. TTS-reported tick bites overlap almost exactly with CDC-confirmed LD cases in county-level and eco-region analyses. In one case, in a county in which TTS respondents did not overlap with human cases, tick encounter reports did overlap with a positive canine county.

The findings show clear patterns which, under other circumstances, could be erroneously interpreted as simple indicators of overlaps in highly populated areas, whereby the more people, the more tick bite reports. However, this exploratory study found that Montague County had the highest number of self-reports of tick bites by survey respondents who reported having LD. Montague County has a population of 19,500. Bowie County, with a population of less than 100,000, in rural East Texas, is third in tick bite reports by LD survey respondents. TTS respondents diagnosed with LD, either clinically or via serological testing, follow an almost exact pattern of tick encounters in the eastern, central, and southern counties in Texas, as well as one Texas Panhandle county, Potter County. The lines of demarcation are undeniably parallel.

As a vector-borne disease, knowing where LD was contracted—i.e., the patient’s geographic location and environmental factors where the infecting tick bite occurred—is critical for epidemiological tracking and understanding conditions that could help inform diagnosis [35]. The authors state: “Prevention and diagnosis of tickborne diseases are improved by access to current and accurate information on where medically important ticks and their associated human and veterinary pathogens are present, their local abundance or prevalence, and when ticks are actively seeking hosts” [35]. The prevalence of tick bites in some locations as opposed to others has meant that, in the U.S., tick-borne illnesses have come to be considered endemic to some states. Accordingly, location is important for surveillance and to aid in potential diagnosis [35], particularly in disaggregated form at the county or ecosystem level. Some non-endemic states may host LD or tick-endemic counties, but surveillance is difficult, as a recent survey reported that less than half of vector-borne disease professionals working in public health were engaged in active tick surveillance [34]. Indeed, further study is needed regarding the location–incidence relationship in terms of contracting LD—information that can be even more useful in states perceived as non-endemic.

If clinically diagnosed patients with known tick bites overlap with CDC-positive cases, further study is indicated to examine geographic parallels. Findings of LD and known tick bites in non-endemic areas also highlight the need for possible follow-up testing and further surveillance—particularly by ecosystem, as ecosystems are not subject to superimposed (non-natural/physical) geographic boundaries, such as county lines. Self-reported cases, as in the TSS, supported by known tick bite encounters in CDC-positive eco-regions, provide evidence that patient-reported, clinically diagnosed LD and CDC-confirmed cases are strongly linked. Adding canine-positive cases by geographic areas in comparison to clinical cases provided further indications that LD may overlap in previously unsuspected patterns. The importance of disease risk mapping across multiple indicators, such as human and canine disease, as well as noting the areas in which ticks have been found to carry disease, may lead to more meaningful public health surveillance.

### 4.2. Limitations

This study reveals the utility of surveying clinically diagnosed and self-reported LD patients as an important means for informing the medical community and policymakers regarding the location of tick bite incidents and symptoms. Beyond providing information on symptoms and personal experiences, if self-reports match officially confirmed cases and veterinary reports, healthcare providers, researchers, and policymakers all are afforded valuable evidence of possible causative links of LD and co-infections among those who may or may not be CDC-positive under the two-tier serological guidelines. This study also suggests that the perception of “non-endemic” states that contain endemic counties requires reconsideration and further evaluation.

This study employs multiple proxies as indicators of human tick-borne disease risk. However, the convenience sample reported tick bites in some areas that are more populated and where survey respondents may simply have access to the Internet and are possibly more aware of disease risk in Texas. Our population density analysis of official CDC cases indicates that this limitation is unlikely. Further research should engage comparison mapping on a national level.

Another possible limitation of this study includes potential information bias, where survey participants in search of diagnosis or validation could perceive their condition as attributable to a tick bite, when the illness has another cause. The sample size is also small. It is unlikely, however, even under these limitations, that tick bite recall would randomly overlap with CDC and canine reports.

Furthermore, the study did not include county maps of reported or established *I. scapularis* tick populations. The CDC relies on tick collection and testing, which suggests that not all counties with infected ticks will appear on a CDC map. Future research should employ the CDC’s map of established and estimated *I. scapularis* tick populations for better informed analyses. Furthermore, the global distribution of vector-borne diseases demonstrates the need for GIS and remote sensing applications to provide public health data on a micro level. Spatial and temporal analysis in a recent study in Denmark, for example, demonstrated differences among controls and LD patients’ proximity to forests [36]. Spatial and temporal patterns can provide more fine-grained public health data, which can lead to improved prevention efforts and alert medical practitioners in local areas.

## 5. Conclusions

Respondents to the online Texans and Ticks Survey who reported a diagnosis of Lyme disease provided county-level data on personal tick bite encounters. Tick bite location was mapped to CDC human cases of Lyme disease, canine tick-borne diseases, and by eco-regions. Self-reported patient experiences are promising for informing public health policy and in offering insights into illnesses with limited diagnostic support. Given the number and consistent overlap of TTS respondents to human and canine tick-borne disease reports, our findings suggest that the thematic multi-layer mapping of LD patients with tick bite location recall may serve as an indicator for human disease risk in areas often perceived to be non-endemic. The TTS study underscores the need for more granular-level mapping of LD and integration of local and demographic information, such as environmental factors including eco-region and data on population density and urbanization.

## Figures and Tables

**Table 1:** *Ixodes scapularis* pathogens and human disease.

Disease	Pathogen(s)	Life Stages Infected
Anaplasmosis	*Anaplasma phagocytophilum*	Nymphs, Adults
Babesiosis	*Babesia microti*	Nymphs, Adults
*Borrelia miyamotoi* disease	*Borrelia miyamotoi*	Larvae, Nymphs, Adults
Ehrlichiosis	*Ehrlichia muris eauclairensis*	Nymphs, Adults
Lyme disease	*Borrelia burgdorferi* sensu stricto, *Borrelia mayonii*	Nymphs, Adults
Powassan virus disease	Powassan virus (lineage II/deer tick lineage)	Larvae, Nymphs, Adults

Centers for Disease Control. Surveillance for Ixodes scapularis and pathogens found in this tick species in the United States.

**Table 2:** TTS respondent characteristics.

Age (N = 95)		
18–30	19	20%
31–45	25	26%
46–60	35	37%
A family member, 17 or younger	7	7%
older than 60 years of age	9	9%
*p* < 0.001		
Occupation (N = 95)		
Not working	32	34%
Student	20	21%
Work Outdoors	6	6%
Work indoors	31	33%
Work indoors, but spend a lot of time outside	18	19%
Diagnostic Type (N = 95)		
Clinical diagnosis by a doctor	46	48%
Western Blot with 5 or more bands, i.e., “CDC Positive”	22	23%
Western Blot with some bands positive, Not “CDC Positive”	33	35%
IGenX or other speciality lab	39	41%
Tick Bite Encounter Location		
Recalled a tick bite	43	45%
*p* = 0.4119		
Symptoms Experienced over the Last Five Years (N = 92)		
Extreme or unusual fatigue	84	91%
Brain fog or concentration problems	84	91%
Headaches	82	89%
Neck or back pain	82	89%
Depression or anxiety	80	87%
Flu-like illness	79	86%
Other unusual or ongoing symptoms	78	85%
Joint Pain or Swelling/Migrating Joint Pain	77	84%
Gastrointestinal/stomach problems	76	83%
Night sweats	71	77%
Fever	62	67%
Rash	57	62%

## Data Availability

Data are available upon request.
